# The Effect of Weekly Set Volume on Strength Gain: A Meta-Analysis

**DOI:** 10.1007/s40279-017-0762-7

**Published:** 2017-07-28

**Authors:** Grant W. Ralston, Lon Kilgore, Frank B. Wyatt, Julien S. Baker

**Affiliations:** 1 Applied Physiology Research Laboratory, Institute for Clinical Exercise and Health Science, School of Science and Sport, University of the West of Scotland, Hamilton, Scotland, UK; 2 Kilgore Academy, Azle, TX USA; 3 Forth Valley College, Falkirk, Scotland, UK; 4 Department of Athletic Training and Exercise Physiology, Midwestern State University, Witchita Falls, TX USA

## Abstract

**Background:**

Strength training set organisation and its relationship to the development of muscular strength have yet to be clearly defined. Current meta-analytical research suggests that different population groups have distinctive muscular adaptations, primarily due to the prescription of the strength training set dose.

**Objectives:**

We conducted a meta-analysis with restrictive inclusion criteria and examined the potential effects of low (LWS), medium (MWS) or high weekly set (HWS) strength training on muscular strength per exercise. Secondly, we examined strength gain variations when performing multi-joint or isolation exercises, and probed for a potential relationship between weekly set number and stage of subjects’ training (trained versus untrained).

**Methods:**

Computerised searches were performed on PubMed, MEDLINE, SWETSWISE, EMBASE and SPORTDiscus™ using the terms ‘strength training’, ‘resistance training’, ‘single sets’, ‘multiple sets’ and ‘volume’. As of September 2016, 6962 potentially relevant studies were identified. After review, nine studies were deemed eligible per pre-set inclusion criteria. Primary data were pooled using a random-effect model. Outcomes for strength gain, strength gain with multi-joint and isolation exercise were analysed for main effects. Sensitivity analyses were calculated for several subgroups by separating the data set and by calculation of separate analyses for each subgroup. Heterogeneity between studies was assessed using the Cochran *Q* and *I* ^2^ statistics.

**Results:**

Pre- versus post-training strength analysis comprised 61 treatment groups from nine studies. For combined multi-joint and isolation exercises, pre- versus post- training strength gains were greater with HWS compared with LWS [mean effect size (ES) 0.18; 95% CI 0.06–0.30; *p* = 0.003]. The mean ES for LWS was 0.82 (95% CI 0.47–1.17). The mean ES for HWS was 1.01 (95% CI 0.70–1.32). Separate analysis of the effects of pre- versus post-training strength for LWS or MWS observed marginally greater strength gains with MWS compared with LWS (ES 0.15; 95% CI 0.01–0.30; *p* = 0.04). The mean ES for LWS was 0.83 (95% CI 0.53–1.13). The mean ES for MWS was 0.98 (95% CI 0.62–1.34). For multi-joint exercises, greater strength gains were observed with HWS compared with LWS (ES 0.18; 95% CI 0.01–0.34; *p* = 0.04). The mean ES for LWS was 0.81 (95% CI 0.65–0.97). The mean ES for HWS was 1.00 (95% CI 0.77–1.23). For isolation exercises, greater strength gains were observed with HWS compared with LWS (ES 0.23; 95% CI 0.06–0.40; *p* = 0.008). The mean ES for LWS was 0.95 (95% CI 0.30–1.60). The mean ES for HWS was 1.10 (95% CI 0.26–1.94). For multi-joint and isolation exercise-specific one repetition maximum (1 RM), marginally greater strength gains were observed with HWS compared with LWS (ES 0.14; 95% CI −0.01 to 0.29; *p* = 0.06). The mean ES for LWS was 0.80 (95% CI 0.47–1.13). The mean ES for HWS was 0.97 (95% CI 0.68–1.26).

**Conclusion:**

This meta-analysis presents additional evidence regarding a graded dose–response relationship between weekly sets performed and strength gain. The use of MWS and HWS was more effective than LWS, with LWS producing the smallest pre- to post-training strength difference. For novice and intermediate male trainees, the findings suggest that LWSs do not lead to strength gains compared with MWS or HWS training. For those trainees in the middle ground, not a novice and not advanced, the existing data provide a relationship between weekly sets and strength gain as set configurations produced different pre- to post-training strength increases. For well trained individuals, the use of either MWS or HWS may be an appropriate dose to produce strength gains.

## Key Points

**Table:** 

Medium (MWS) or high weekly set (HWS) strength training is marginally more effective in producing strength gains than low weekly set (LWS) strength training.
The use of either MWS or HWS training may be an appropriate dose to produce strength gains for well trained males.
For male trainees at novice and intermediate level, MWS training is more effective than LWS training for both multi-joint and isolation exercises.
For those trainees in the middle ground (not a novice or advanced), the existing data provide evidence of a pre- to post-graded set dose–response relationship in strength for both multi-joint and isolation exercises.

## Introduction

### Rationale

Strength training is the most popular form of exercise for developing musculoskeletal strength [1]. The physiological adaptations of a strength training programme can result in increased strength, muscular hypertrophy, connective tissue thickness, increased fat-free mass and improved motor functioning [2]. Over the last decade, studies have demonstrated greater strength gains with multiple sets (MS) per exercise [3–5] than when performing one set. This has been reinforced by several meta-analyses that advocate the use of MS to promote greater strength gains in both trained and untrained subjects [6–9].

The meta-analysis by Rhea et al. [6] reported significant magnitude in strength gains between trained and untrained groups. The effective size (ES) for both men and women, regardless of training status, were similar and the dose–response curves comparable for all age ranges. Rhea and colleagues’ meta-analysis [6] reported a significant difference between one and three sets (ES = 2.3 bench press and ES = 6.5 leg press, respectively). The one-set bench press pre-to-post had a reported strength increase of 20% compared with the three-set bench press that resulted in a strength increase of 33%. The pre- to post-leg press strength resulted in a strength increase of 25.4% for one set compared with a strength increase of 52.1% with three sets. The pre- to post-leg press strength difference was more than eight times greater than a large ES (6.5), which presents an extraordinary result in the scientific literature that has not been reproduced.

The meta-analysis by Peterson et al. [9] concluded that competitive athletes should perform eight sets per muscle group to increase muscular strength; however, limited evidence supported this within the review. The conclusions were inconsistent due to the small number of ESs in the eight-set group, creating unpredictable patterns of evidence when analysing specific one repetition maximum training percentages (% 1RM). The ES for training at 70% 1RM indicated no impact upon subject’s strength. When the percentage 1RM loads were increased to 75% 1RM and 85% 1RM, a large ES was reported but also a very large standard deviation. No physiological mechanism or theory can validate why a variation of 5% 1RM can result in such large strength differences. Peterson et al. [9] provided limited descriptions concerning the volume intensity and only included the mean ES that demonstrated maximal strength gains. No data supporting loading or strength differences in kilograms were provided to allow informed decisions on the evidence to be made.

Wolfe et al. [7] meta-analysis reported that resistance training (RT) programmes that lasted between 17 and 40 weeks did not generate significantly higher ESs in comparison with those between 6 and 15 weeks. This suggests that training progression is linear and there was no reported difference between 6–15 and 17–40 weeks in strength gain. The data indicated that trained individuals performing multiple sets generated greater increases in strength (*p* ≤ 0.001) and that a single-set programme may be used for untrained individuals after the initial 6–15 weeks. The untrained individuals were reported to have similar gains in strength as that of trained individuals when performing multiple sets. The difference in mean value for the set number was statistically significant when allowing for the effects of changes in programme duration (*p* ≤ 0.005). Specifically, multiple-set programmes produced larger increases in strength compared with single sets (*p* ≤ 0.002) when RT exercises were performed to subjects’ physical failure versus subjects’ own perceived end. The main conclusion of the Wolfe et al. [7] analysis was no significant differences for the subjects’ physical failure versus subjects’ own perceived end and the number of sets performed (*p* ≤ 0.052) when trained and untrained subjects were separated by training status.

Krieger [8] compared the effects of single versus multiple sets of exercises on strength, using a hierarchical random effects linear mixed model meta-regression. The analysis consisted of 14 studies (440 participants), with 30 treatment groups and a total of 92 ESs. The results indicated that multiple sets were associated with a larger ES than one set. When the dose–response model was further analysed, there was a drift towards two to three sets per exercise compared with one set. No significant difference was reported between one set per exercise and four to six sets per exercise or between two to three sets per exercise and four to six sets per exercise. Krieger [8] concluded that two to three sets per resistance exercise was associated with 46% greater strength gains than one set in both trained and untrained subjects.

Garber et al. [10] constructed the American College of Sports Medicine (ACSM) position stand that provides guidance on the individual prescription of exercise to apparently healthy adults of all ages. In that position stand, Garber et al. [10] cited four meta-analytical studies that contain contradicting recommendations [6–9]. Otto and Carpinelli [11] questioned the validity of the Rhea et al. [6] meta-analysis due to the inclusion of the Rhea et al. [12] study. The Rhea et al. [12] study had a post-training bench press ES three to four times superior to the pre-training measurement in one and three sets with no confidence intervals included with the ESs. Another Rhea et al. [13] meta-analysis of 140 studies with a total of 1433 ESs was conducted to identify any dose–response relationship. The ESs demonstrated different responses based on training status of the trainees. Untrained individuals experienced maximal strength gains by training each muscle group 3 days·week^−1^ with four sets per muscle group. Trained individuals should train at a frequency of 2 days·week^−1^ with four sets per muscle group. The analysis of Rhea et al. [13] supports the theory of progression in RT for strength development. Carpinelli [14], however, has questioned the meta-analytical evidence and the methods used to select the studies, extract the data and assess study validity, which were not clearly described.

The existing consensus regarding the association of sets of exercises completed and strength development remains controversial. This is due to the lack of a controlled and quantified relationship between strength training variables presented in the primary research literature. The inclusion of low-quality studies within a meta-analysis can introduce spurious conclusions regarding strength outcomes, as variations in the exercises included in studies, variation in subject characteristics, and variance in experimental programme design may affect reliability and accuracy. As a consequence of previous imprecision and poor experimental control, conclusions with respect to set number and strength gain are likely not reflective of the specific adaptations resulting from imposed physiological stress.

### Objectives

Although meta-analyses regarding the effects of set volume on strength have been published [6–9], none limited their analyses to measures of strength by weekly set volume with restricted subject pools adequately. The purpose of this paper was to conduct a meta-analysis on the effects of low (LWS), medium (MWS) or high weekly set (HWS) strength training on male muscular strength per exercise. A secondary purpose was to establish if multi-joint exercises produce a different strength gain profile when compared with single joint exercises by specific weekly set training. A third purpose was to provide a perspective on developing muscular strength across stages of training progression in male trainees. Based on previous meta-analytic data [8], we hypothesised that there would be a pre- to post-training graded dose–response relationship in strength, with higher weekly set training promoting superior strength results.

## Methods

### Eligibility Criteria

Included studies were selected and initially coded according to the following criteria: (a) RT programme lasting a minimum of 4 weeks; (b) training at least one major muscle group—quadriceps (vastus medialis, vastus intermedius, vastus lateralis, rectus femoris), hamstrings (bicep femoris, semitendinosus, semimembranosus), pectoralis major, latissimus dorsi, deltoids (anterior, lateral, posterior), biceps or triceps; (c) adult male subjects aged 18–60 years; (d) compared single versus multiple sets per exercise; (e) all subjects free from muscular skeletal, or orthopaedic injuries, or physical limitations; (f) pre- to post-1 RM measurement of muscular strength; (g) subject’s descriptive characteristics included in report (height, weight, training status and training experience); (h) sufficient data to determine sets and intensity of exercise and to calculate ESs. Randomised controlled trials (RCTs) and randomly assigned trials (RANs) that observed the intervention programmes using stratified single- versus multiple-set dosages were used for this analysis. RCTs represent a more rigorous method for determining a cause–effect relationship between treatment and outcome. RAN allocation ensures no systematic differences between intervention groups; however, no control group is included within this research design that may impact upon the assessment of outcomes.

### Information Sources

Computerised searches were performed to generate citation lists from the following databases: PubMed, MEDLINE, SWETSWISE, EMBASE, SPORTDiscus™. The period of search history examined was comprehensive to September 2016. Additional relevant studies were identified by hand searching and cross-referencing of key journals, reference lists and other sources. Relevant descriptive terms used to retrieve studies in English were ‘strength training’, ‘resistance training’, ‘single sets’, ‘multiple sets’ and ‘volume’. Boolean operators, including AND, OR and NOT, were used to focus literature searches.

### Study Selection

The literature searches were limited to training studies involving humans. This resulted in retrieval of papers published from 1987 through to September 2016 in which single-set versus multiple-set interventions were compared, in both untrained and trained subjects. Abstracts and citations from scientific conferences and studies published in foreign language journals were excluded.

### Data Collection Process

All calculations were conducted using a Microsoft Excel (Microsoft, Redmond, WA, USA) spreadsheet containing data extracted from each publication. In addition, Review Manager (RevMan) version 5.3.5 was used for all statistical analyses and forest plots. Cochran *Q* statistic [15] was used to assess heterogeneity between studies. Heterogeneity refers to the existence of variation between studies on the main effects being evaluated. This is an appropriate test for larger meta-analyses and uses the sum of squared deviations of the specific estimates derived from the pooled estimate and weights the contribution of each study. The *p* values were achieved by comparing the *Q* statistic with a *Χ* ^2^ distribution with *k* ^−1^ degrees of freedom, where *k* represents the number of included studies. In addition, the *I* ^2^ statistic was used to assess heterogeneity, with an *I* ^2^ > 50% used to indicate heterogeneity. RT programme effects for muscular strength were calculated for each included study following coding of pre-to-post changes and standard deviations (SDs). The mean difference (MD) or change in post-intervention mean was calculated by subtracting the baseline from post-intervention values for all strength outcome measures. Change in the SD of post-intervention outcomes was calculated using RevMan (version 5.3.5). Data were required to be either (1) mean and SDs (pre- and post-intervention), (2) 95% confidence interval (CI) data for pre- to post-intervention change for each group, or when this was unavailable, (3) actual *p* values for pre- to post-intervention change for each group, or if only the level of statistical significance was available, (4) default *p* values (e.g. *p* ≤ 0.05 becomes *p* ≤ 0.49, *p* ≤ 0.01 becomes *p* ≤ 0.0099 and *p* ≤ not significant becomes *p* ≤ 0.05). A random-effects inverse variance (IV) was used with the effects measure of MD.

The analysis of ES was conducted with a random-effects model estimated using the DerSimonian and Laird method [16]. A random-effects model is incorporated when the assumption is that the effect across studies is randomly situated about a central value.

For each of the two measures, forest plots were generated to demonstrate the study-specific pre- to post-training strength differences and ESs, within the respective 95% CIs. Combining estimates then allowed for assessment of a pooled effect, in which the reciprocal of the sum of two variances was accounted for, including (1) the estimated variance associated with the study and (2) the estimated component of variance due to the variation between studies. Visual inspection of forest plots for each performance measure against its standard error was included to account for the ‘file drawer problem’, the potential effect of published studies being intrinsically biased due to a greater probability of significant results.

Separate meta-regressions on ESs were performed with the following moderator variables: total sets per RT exercise per week as a continuous variable; total sets per multi-joint exercise per week categorised as LWS (≤5), MWS (5–9) or HWS (≥10); total sets per isolation exercise per week categorised as LWS (≤5), MWS (5–9) or HWS (≥10). In the regression model, mean differences in ES were calculated for each study to give a study-level ES for the difference between LWS, MWS and HWS to allow for the generation of forest plots. A sensitivity analysis was conducted to identify the presence of highly influential studies which might bias the analysis. This was performed for each model by removing one study at a time and then examining the weekly set volume predictor. Influential studies were identified and removed if they resulted in a change from significant (*p* ≤ 0.10) to nonsignificant (*p* ≥ 0.10), or vice versa, or if removal caused a large change in the magnitude of the coefficient.

Articles deemed to meet inclusion criteria were obtained and examined by the primary reviewer. In the case of inadequate information from selected manuscripts, the secondary reviewer confirmed satisfaction or non-satisfaction of inclusion criteria. In each study, the ES for the intervention was calculated as the difference between the means of the pre-test and post-test at the end of the RT intervention. The study-specific weight was derived as the inverse of the square of the respective standard errors. The ESs of ≤0.2, ≤0.5, ≤0.8 and ≥0.8 were considered trivial, small, moderate and large, respectively [17].

## Results

**Fig. 1:**
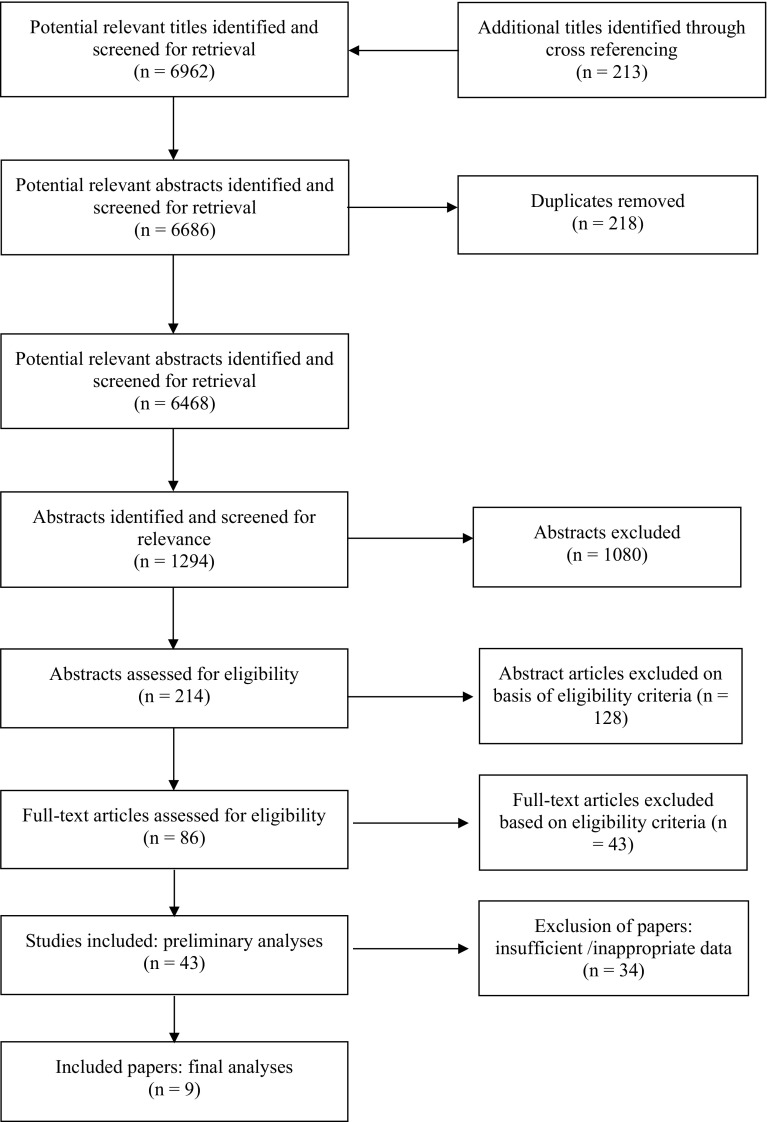
Flow of journal articles through the systematic review process

The flow of article search and selection is depicted in Fig. 1, from ‘potentially relevant’ to final article inclusion.

### Study Selection

The preliminary search yielded 6962 relevant abstracts and citations. The full text of 86 articles was deemed to meet inclusion criteria (Table 1). A total of nine studies with 223 subjects were deemed eligible per the inclusion criteria [13, 18–25]. Articles included in the analysis had publication dates ranging from 1987 to 2016. The experimental design of included studies had a random assignment of treatment conditions (RAN) and randomised control groups (RCT). The training status of subjects included in the nine studies was trained (*n* = 4) and untrained (*n* = 5) males (Table 2).

**Table 1:** Meta-analysis inclusion and exclusion criteria

Inclusion criteria	Exclusion criteria
Strength assessment of one or more muscle groups used (isolation exercises, e.g. leg extension with stress gauge)	Legal or illegal ergogenic aids or supplementation has been used during interventions
Minimum duration of training intervention is 4 weeks, longitudinal studies would be preferred (>12 weeks)	Variation within the training order throughout the weeks
Familiarisation prior to baseline testing with inclusion of a minimum of 1 week ‘washout’ of other RT training if applicable	No quasi-RCT or narrative studies/reviews to be included
Preferred if control group included within research design with subjects randomly assigned to groups	Mixed-sex studies
RT programme supervised with the RT intervention of similar order and if applicable inter-set recovery periods standardised for multiple sets	Male subjects with age >60 years
Conducted warm up is standardised between intervention groups and if comparing one set to multiple sets establish working sets only	
Subjects trained to volitional exhaustion with appropriate criteria regarding training intensity	
Comparison of 1 set to 2, 3, 4, 4+	

*RCT* randomised controlled trials, *RT* resistance training

### Study Characteristics

In total, nine studies provided data on 223 male subjects (Table 2). The mean age of the subjects was 23.4 years (±2.18 years). The length of training ranged from 8 to 26 weeks (mean 11.11 ± 5.7 weeks), frequency ranged from two to four times per week (mean 2.8 ± 0.32 per week) and the exercise intensity ranged from 73.5 to 85% of the 1RM (mean 78.2 ± 4.1% 1RM). The number of sets reported ranged from one to 12 sets (mean 3.14 ± 2.63 sets). The within-group number of repetitions performed ranged from three to 18 (mean 8.8 ± 1.6 repetitions).

**Table 2:** Study and subject characteristics

Study	Design	Status	*N*	Age range, y	Frequency per wk	Duration, wk	Sets	Reps	Training loads,% 1RM (mean ± SD)	Outcomes 1RM strength
Rhea et al. [12]	RAN	T	16	20–22	3	12	1/3	8–12	67–80^a^ (73.5 ± 6.5)	Bench press/leg press
Ostrowski et al. [18]	RAN	T	27	18–29	4	10	1/2/4	7–12	67–83^a^ (75 ± 8)	Bench press/squat
Paulsen et al. [19]	RAN	UT	18	20–30	3	6	1/3	7	83^a^	Bench press/squat
Marshall et al. [20]	RAN	T	32	Young males	2	6	1/4/8	8	80^a^	Back squat
Baker et al. [21]	RAN	T	16	18–21	3	8	1/3	6	85^a^	Bench press/shoulder press
Radaelli et al. [22]	RCT	UT	48	23.5–25.3	3	26	Con/1/3/5	8–12	67–80^a^ (73.5 ± 6.5)	Bench press/lateral pull-down/shoulder press/leg press
Bottaro et al. [23]	RAN	UT	24	19–25.4	2	12	1/3	8–12	67–85^a^ (76 ± 9)	Knee extension Elbow extension
Reid [24]	RAN	UT	34	18–35	3	8	1/2/3	3–18	63–93^a^ (78 ± 21.2)	Elbow flexion/elbow extension/knee flexion/knee extension/shoulder flexion/shoulder extension
Sooneste et al. [25]	RAN	UT	8	22.9–27.1	2	12	1/3	8	80	Seated preacher curl
Total/mean ± SD			223	23.4 (±2.18)	2.8 (±0.32)	11.11 (±5.7)	3.14 (±2.63)	8.8 (±1.6) reps	78.2 (± 4.1)	

*% 1RM* percentage of one repetition maximum, *1RM* one repetition maximum, *Con* control group *N* number of subjects, *per wk* number of days trained per week, *RAN* randomly assigned trial, *RCT* randomised controlled trial, *Reps* repetitions, *SD* standard deviation, *T* trained, *UT* untrained, *wk* weeks, *y* years

^a^Estimated reps at% of 1RM [42]

### Sensitivity Analysis

**Fig. 2:**
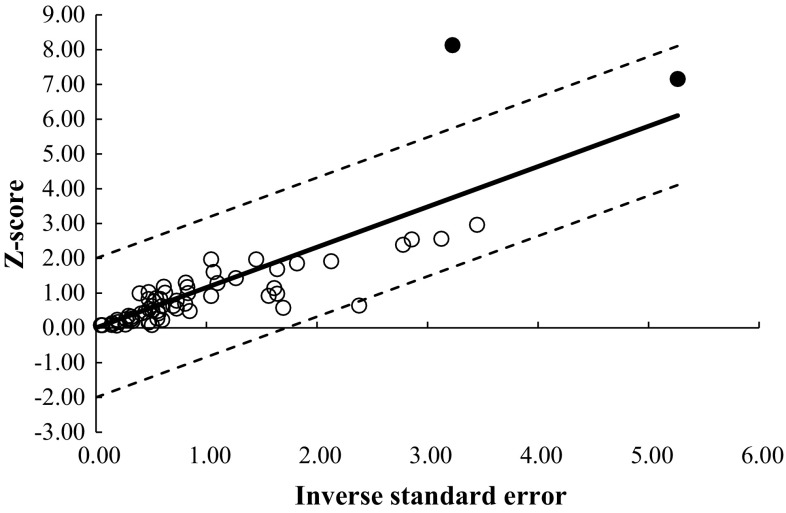
Galbraith plot used to examine study heterogeneity (pre- vs post-intervention strength change). Each *open circle* represents one pre- vs post-intervention study datum. Two pre- vs post-intervention study data of Reid [24] identified as outliers (*solid filled black circles*)

**Fig. 3:**
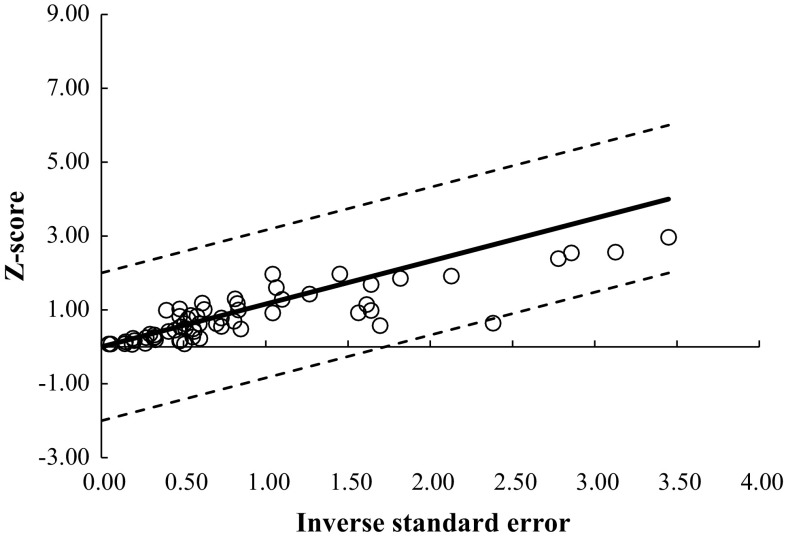
Galbraith plot with the removal of two pre- vs post-intervention study outliers (Reid [24]). *Each open circle* represents one pre- vs post-intervention study datum

**Fig. 4:**
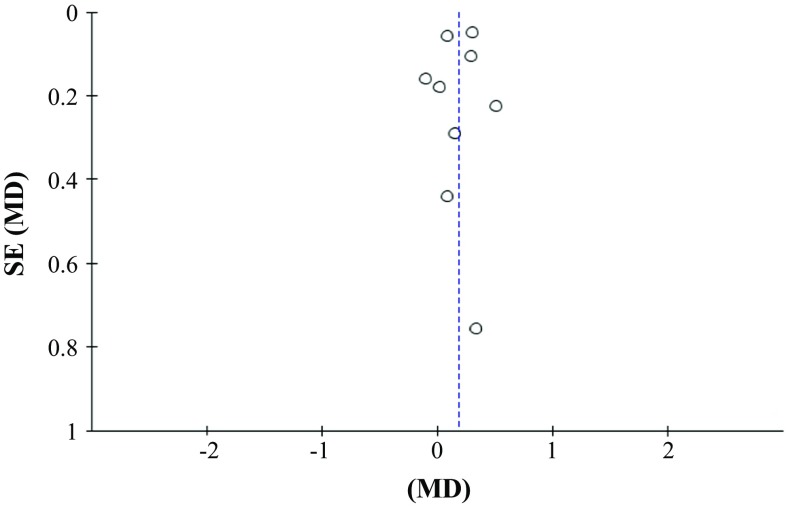
Funnel plot of standard error (*SE*) by mean difference (*MD*) for assessment of publication bias. *Each open circle* denotes a study included in the meta-analysis. The *dashed vertical line* represents the overall effect calculated with the random-effects model

To investigate for study heterogeneity, Galbraith plots were used to identify any potential outliers (Fig. 2). Examination Galbraith plots revealed that Reid [24] elbow extension and shoulder flexion data were influential (Fig. 3). Removal of Reid [24] data reduced the impact of the weekly number of sets on strength gain (Table 3). Trim and fill funnel plots and Egger’s tests were performed to assess for publication bias of literature in all comparison models. The shape of the funnel plot did not reveal any evidence of obvious asymmetry (Fig. 4). Results from an Egger’s test (*p* = 0.393) confirmed no evidence of publication bias.

**Table 3:** Pre- versus post-intervention strength analysis of multi-joint exercise

Study	*N*	Age range (y)	Frequency/duration	Testing modality	Sets (reps)	Training loads	Weekly sets per exercise	Pre- vs post-intervention kg, mean ± SD	Pre- vs post-intervention% strength difference, kg (mean)	*p* value (pre- vs post-intervention)	ES
Rhea et al. [13]	16	19–23	3 per wk For 12 wk	LP	1 (8–12)	8–12 RM	LWS	269.0 ± 16.8 vs 337.2 ± 69.0	68.2 (25.4)	≤0.05^a^	1.36
				LP	3 (8–12)		HWS	225.9 ± 25 vs 343.5 ± 89.9	117.6 (52.1)	≤0.05^a^	1.78
Rhea et al. [13]	16	19–23	3 per wk For 12 wk	BP	1 (8–12)	8–12 RM	LWS	64.2 ± 8.9 vs 76.7 ± 28.0	12.5 (19.5)	?	0.60
				BP	3 (8–12)		HWS	66.8 ± 7.3 vs 85.5 ± 20.8	18.7 (21.9)	?	1.20
Ostrowski et al. [18]	27	18–29	4 per wk For 10 wk	BP	1 (7–12)	7–12 RM	LWS	89.7 ± 11.1 vs 93.3 ± 10.9	3.6 (4.0)	?	0.33
				BP	2 (7–12)		MWS	90.8 ± 9.4 vs 95.3 ± 9.5	4.5 (5.0)	?	0.48
				BP	4 (7–12)		HWS	83.1 ± 9.7 vs 84.7 ± 10.3	1.6 (1.9)	?	0.16
Ostrowski et al. [18]	27	18–29	4 per wk For 10 wk	Sq	1 (7–12)	7–12 RM	LWS	134 ± 28.4 vs 144 ± 27.8	10 (7.5)	≤0.05^a^	0.36
				Sq	2 (7–12)		MWS	146 ± 23.1 vs 154 ± 20.7	8 (5.5)	≤0.05^a^	0.36
				Sq	4 (7–12)		HWS	121 ± 20.7 vs 135 ± 16.3	14 (11.6)	≤0.05^a^	0.75
Paulsen et al. [19]	18	20–30	3 per wk For 6 wk	Sq	1 (7)	7 RM	LWS	129.5 ± 20.6 vs 147 ± 21.2	17.5 (13.5)	≤0.01^b^	0.84
				Sq	3 (7)		MWS	122.5 ± 29 vs 149.4 ± 29.0	26.9 (22.0)	≤0.01^b^/≤0.05^c^	0.93
Paulsen et al. [19]	18	20–30	3 per wk For 6 wk	BP	1 (7)	7 RM	LWS	74.8 ± 7.0 vs 82.3 ± 8.3	7.5 (10)	≤0.01^b^	0.98
				BP	3 (7)		MWS	77.8 ± 11.3 vs 85.0 ± 12.9	7.2 (9.3)	≤0.01^b^/≤0.05^c^	0.59
Marshall et al. [20]	32	Young males	2 per wk For 6 wk	BSq	1 (8)	8 RM	LWS	149 ± 7.8 vs 165.5 ± 9.2	16.5 (11.1)	≤0.05^a^	0.61
				BSq	4 (8)		MWS	157.3 ± 12.2 vs 178.2 ± 11.8	20.9 (13.3)	≤0.05^a^	0.55
				BSq	8 (8)		HWS	162.0 ± 11.8 vs 194.9 ± 14.3	32.9 (20.3)	≤0.05^a^	0.84
Baker et al. [21]	16	18–21	3 per wk For 8 wk	BP	1 (6)	6 RM	LWS	67.2 ± 11.5 vs 79.2 ± 12.4	11.9 (17.9)	≤0.05 one tailed^a^	1.00
				BP	3 (6)		MWS	68.5 ± 13.4 vs 80.5 ± 9.8	9.8 (17.5)	≤0.05 one tailed^a^	1.02
Baker et al. [21]	16	18–21	3 per wk For 8 wk	SP	1 (6)	6 RM	LWS	42.1 ± 7.3 vs 53.8 ± 7.6	11.7 (27.8)	≤0.05 one tailed^a^	1.57
				SP	3 (6)		MWS	42.7 ± 5.0 vs 52.0 ± 6.4	9.4 (21.8)	≤0.05 one tailed^a^	1.62
Radaelli et al. [22]	48	23.5–25.3	3 per wk For 6 mo	BP	Con (0)	8–12 RM		68.3 ± 11.4 vs 64.4 ± 8.8	–2.8 (–1.8)	?	–0.38
				BP	1 (8–12)		LWS	64.5 ± 9.5 vs 73.2 ± 9.9	8.7 (15.7)	≤0.05^a, c^	0.90
				BP	3 (8–12)		MWS	73.4 ± 9.4 vs 86.1 ± 8.4	12.7 (15.5)	≤0.05^a, c, d^	1.42
				BP	5 (8–12)		HWS	89.6 ± 9.6 vs 99.6 ± 5.5	23.0 (12.9)	≤0.05^a, d^	1.13
Radaelli et al. [22]	48	23.5–25.3	3 per wk For 6 mo	LPul	Con (0)	8–12 RM		60.5 ± 6.8 vs 62.2 ± 6.6	1.7 (2.8)	?	0.25
				LPul	1 (8–12)		LWS	57.9 ± 10.7 vs 68.7 ± 9.5	10.8 (18.7)	≤0.05^a, c, d^	1.07
				LPul	3 (8–12)		MWS	62.5 ± 6.21 vs 70.0 ± 4.76	7.5 (12.0)	≤0.05^a, c, d^	1.36
				LPul	5 (8–12)		HWS	74.2 ± 9.5 vs 86.5 ± 6.5	12.3 (16.6)	≤0.05^a, c, d^	1.51
Radaelli et al. [22]	48	23.5–25.3	3 per wk For 6 mo	SP	Con (0)	8–12 RM		26.1 ± 7.4 vs 29.4 ± 7.6	3.3 (12.6)		0.44
				SP	1 (8–12)		LWS	31.6 ± 7.1 vs 38.7 ± 9.3	7.1 (22.5)	≤0.05^a, d^	0.86
				SP	3 (8–12)		MWS	34.2 ± 7.5 vs 42.3 ± 6.3	8.1 (23.7)	≤0.05^a, c, d^	1.17
				SP	5 (8–12)		HWS	41.5 ± 8.2 vs 56.1 ± 11.9	14.6 (35.2)	≤0.05^a, c, d^	1.43
Radaelli et al. [22]	48	23.5–25.3	3 per wk For 6 mo	LP	Con (0)	8–12 RM		157.8 ± 21.0 vs 155.0 ± 25.0	–2.8 (–1.8)	?	–0.12
				LP	1 (8–12)		LWS	170 ± 34.1 vs 196.7 ± 15.5	3.3 (15.7)	≤0.05^a, d^	1.01
				LP	3 (8–12)		MWS	172.5 ± 30.1 vs 199.2 ± 14.4	26.7 (15.5)	≤0.05^a, c, d^	1.13
				LP	5 (8–12)		HWS	178.5 ± 24.4 vs 201.5 ± 25.4	26.7 (12.9)	≤0.05^a, d^	0.92

*?* data not available, *Con* control group, *BP* bench press, *BSq* back squat, *ES* effect size, *HWS* high weekly sets per exercise (≥10), *kg* kilograms, *LP* leg press, *LPul* lateral pull-down, *LWS* low weekly sets per exercise (≤5), *MWS* medium weekly sets per exercise (5–9), *N* number of subjects, *per wk* number of days trained per week, *Reps* repetitions, *RM* repetition maximum, *SD* standard deviation, *SP* shoulder press, *Sq* squat, *y* years

^a^Significantly greater than prior to training (*p* ≤ 0.05)

^b^Significant differences between groups (*p* ≤ 0.01)

^c^Significant differences between groups (*p* ≤ 0.05)

^d^Statistically significant difference compared with the three-set group (*p* ≤ 0.05)

### Effect of Weekly Set Volume on Multi-Joint and Isolation Resistance Exercise

Pre- to post-intervention strength differences were assessed via meta-analysis for all included studies, then multi-joint exercises were combined into a sub-group analysis and isolation exercises were combined into a separate sub-group analysis. Due to potential heterogeneity, a random effects model was incorporated with *I* ^2^ used to assess each strength measure.

#### Effects of Weekly Set Volume on Combined Multi-Joint and Isolation Exercise

**Fig. 5:**
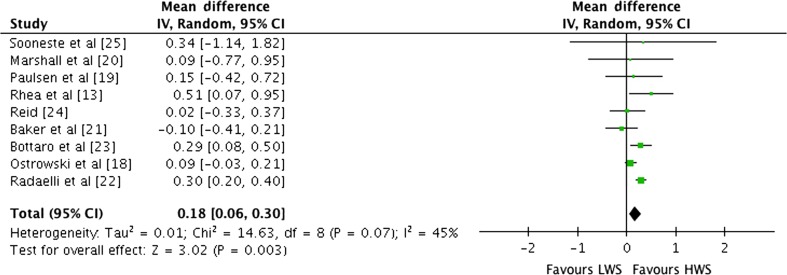
Forest plot of LWS vs HWS (MWS and HWS combined) on multi-joint and isolation exercise by study. The *vertical line* indicates the overall estimate of combined multi-joint and isolation studies mean effect size. The *horizontal line* indicates 95% CI, *squares* indicate estimates, whereas square size is proportional to sample size, and *rhombus* indicates meta-analytically pooled estimates 95% CI. *95% CI* 95% confidence interval, *HWS* high weekly sets per exercise (≥10), *IV* inverse variance, *LWS* low weekly sets per exercise (≤5), *MWS* medium weekly sets per exercise (5–9)

Outcomes for weekly sets categorised as LWS or HWS (MWS and HWS combined) are shown in the forest plot in Fig. 5. The forest plot contains the mean ESs and corresponding CIs for strength gain separated for interventions featuring LWS and HWS as well as the overall effect test and heterogeneity analysis. The pooled mean ES estimates of multi-joint and isolation data (Table 3) comprised 61 treatment groups from nine studies [13, 18–25]. There was moderate heterogeneity detected in the nine studies included in the meta-analysis (*I* ^2^ = 45%). When a random effects analysis was applied, a trivial effect was observed for multi-joint and isolation weekly set outcomes (ES 0.18; 95% CI 0.06–0.30; *p* = 0.003). Pre- to post-intervention strength gain was greater with HWS compared with LWS (ES difference 0.19). The mean ES for LWS was 0.82 (95% CI 0.47–1.17). The mean ES for HWS was 1.01 (95% CI 0.70–1.32).

**Fig. 6:**
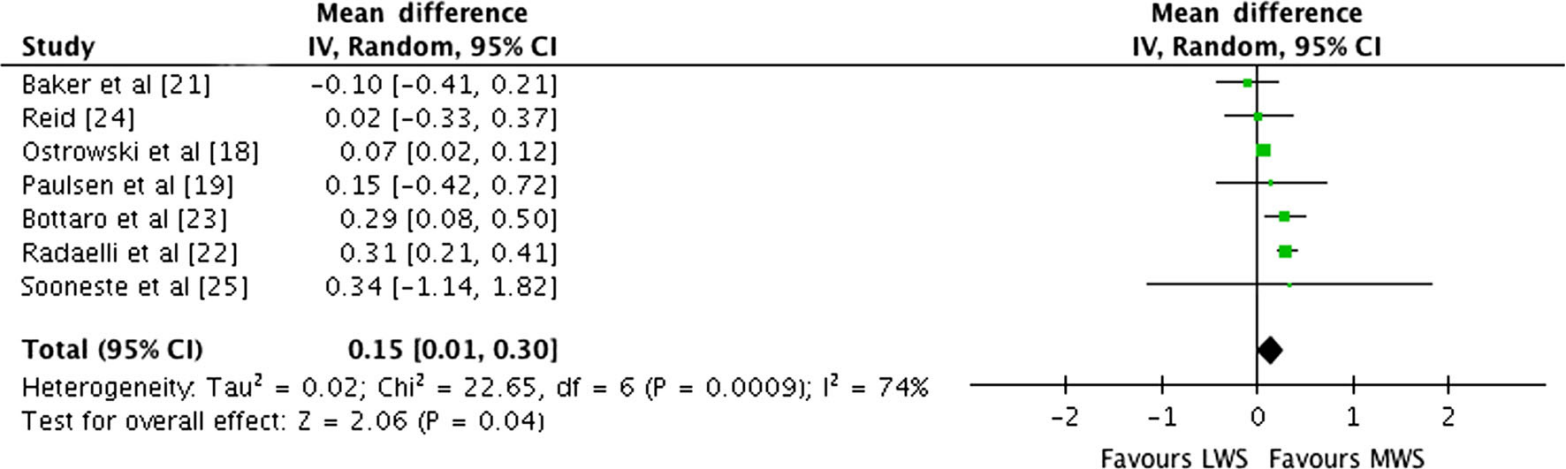
Forest plot of LWS vs MWS on multi-joint and isolation exercise by study. The *vertical line* indicates the overall estimate of combined multi-joint and isolation studies mean effect size. The *horizontal line* indicates 95% CI, *squares* indicate estimates, whereas square size is proportional to sample size, and *rhombus* indicates meta-analytically pooled estimates 95% CI. *95% CI* 95% confidence interval, *IV* inverse variance, *LWS* low weekly sets per exercise (≤5), *MWS* medium weekly sets per exercise (5–9)

Outcomes for weekly sets categorised as LWS or MWS within each study are shown in Fig. 6. There was significant heterogeneity detected in the seven studies (*I* ^2^ = 74%). When a random-effects analysis was applied, a trivial effect was observed (ES 0.15; 95% CI 0.01–0.30; *p* = 0.04). Pre- to post-intervention strength gain was marginally greater with MWS compared with LWS (ES difference = 0.15). The mean ES for LWS was 0.83 (95% CI 0.53–1.13). The mean ES for MWS was 0.98 (95% CI 0.62–1.34). Examination of MWS versus HWS pre-to post-intervention strength differences was not feasible due to limited study data.

#### Effect of Weekly Set Volume on Multi-Joint Exercise

**Fig. 7:**
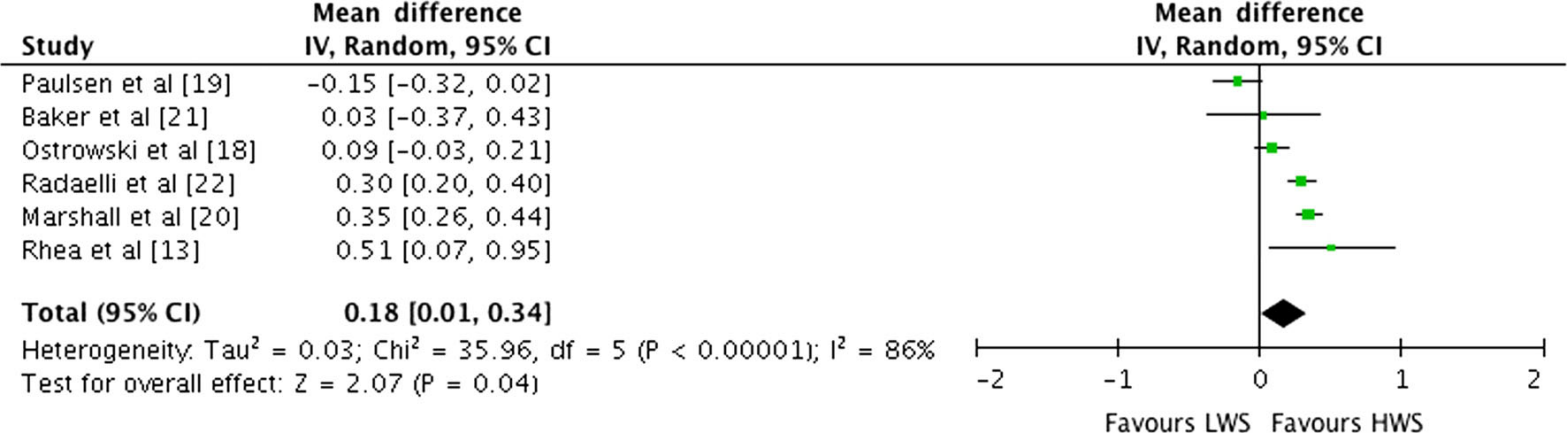
Forest plot of LWS vs HWS (MWS and HWS combined) on multi-joint exercises by study. The *vertical dashed line* indicates the overall estimate of multi-joint studies mean effect size. *Horizontal lines* indicate 95% CI, *squares* indicate estimates, whereas square size is proportional to sample size, and *rhombus* indicates meta-analytically pooled estimates 95% CI. *95% CI* 95% confidence interval, *HWS* high weekly sets per exercise (≥10), *IV* inverse variance, *LWS* low weekly sets per exercise (≤5), *MWS* medium weekly sets per exercise (5–9)

Outcomes for weekly sets categorised as LWS or HWS (MWS and HWS combined) for multi-joint exercises are shown in the forest plot in Fig. 7. The pooled mean ES estimates of multi-joint-only exercise strength data comprised 33 treatment groups from six studies [13, 18–22]. There was significant heterogeneity detected in the six studies included in the meta-analysis of multi-joint-only weekly set outcomes (*I* ^2^ = 86%). When a random effects analysis was applied, a trivial effect was observed (ES 0.18; 95% CI 0.01–0.34; *p* = 0.04). Pre- to post-intervention strength gain was greater with HWS compared with LWS (ES difference 0.19). The mean ES for LWS was 0.81 (95% CI 0.65–0.97). The mean ES for HWS was 1.00 (95% CI 0.77–1.23). Examination of MWS versus HWS pre- to post-intervention strength differences was not feasible due to limited study data.

#### Effect of Weekly Set Volume on Isolation Exercise

**Fig. 8:**
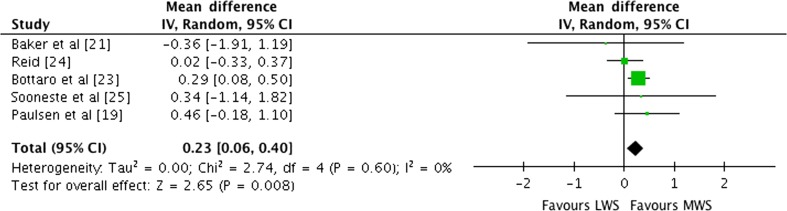
Forest plot of LWS vs MWS on isolation exercises by study. The *vertical dashed line* indicates the overall estimate of isolation studies mean effect size. *Horizontal lines* indicate 95% CI, *squares* indicate estimates, whereas square size is proportional to sample size, and rhombus indicates meta-analytically pooled estimates 95% CI. *95% CI* 95% confidence interval, *IV* inverse variance, *LWS* low weekly sets per exercise (≤5), *MWS* medium weekly sets per exercise (5–9)

Outcomes for weekly sets categorised as LWS or MWS for isolation exercises are shown in the forest plot in Fig. 8. The pooled mean ES estimate of combined isolation exercises (Table 4) comprised 28 treatment groups from five studies [19, 21, 23–25]. There was no heterogeneity detected in the five studies included in the meta-analysis of isolation-only weekly set outcomes (*I* ^2^ = 0%). When a random effects analysis was applied, a small effect was observed (ES 0.23; 95% CI 0.06–0.40; *p* = 0.008). Pre- to post-intervention strength gain was greater with HWS compared with LWS (ES difference 0.15). The mean ES for LWS was 0.95 (95% CI 0.30–1.60). The mean ES for HWS was 1.10 (95% CI 0.26–1.94). Examination of HWS pre- to post-intervention strength differences was not feasible due to limited study data.

**Table 4:** Pre- versus post-intervention strength analysis on isolation exercise

Study	*N*	Age, y [range or mean ± SD]	Frequency/duration	Testing modality	Sets (reps)	Training loads	Weekly sets per exercise	Pre- vs post-intervention kg, mean ± SD	Pre- vs post-intervention% strength difference, kg (mean)	*p* value (pre- vs post-intervention)	ES
Paulsen et al. [19]	18	20–30	3 per wk For 6 wk	KExt	1 (7)	7 RM	LWS	125.8 ± 16.7 vs 144 ± 14.4	18.2 (14.5)	≤0.01^a^	1.17
				KExt	3 (7)		MWS	117.8 ± 13.5 vs 142.5 ± 8.9	24.7 (21.0)	≤0.01^a^/≤0.05^b^	2.16
Paulsen et al. [19]	18	20–30	3 per wk For 6 wk	LC	1 (7)	7 RM	LWS	57.3 ± 9.6 vs 64.8 ± 7.6	7.5 (13.1)	≤0.01^a^	0.87
				LC	3 (7)		MWS	55.9 ± 10.3 vs 65.3 ± 13.1	9.4 (16.8)	≤0.01^a^/≤0.05^b^	0.80
Baker et al. [21]	16	18–21	3 per wk For 8 wk	BC	1 (6)	6 RM	LWS	41.1 ± 5.6 vs 49.5 ± 4.9	8.4 (20.3)	≤0.05 one tailed^c^	1.60
				BC	3 (6)		MWS	43.0 ± 4.5 vs 51.0 ± 7.9	7.8 (18.6)	≤0.05 one tailed^c^	1.24
Bottaro et al. [23]	24	22.2 ± 3.2	2 per wk For 12 wk	KExt	1 (8–12)	8–12 RM	LWS	24.3 ± 3.0 vs 25.3 ± 2.9	1.0 (4.1)	?	0.34
				KExt	3 (8–12)		MWS	20.9 ± 3.2 vs 23.4 ± 2.3	2.4 (12.0)	≤0.05^c^	0.90
Bottaro et al. [23]	24	22.2 ± 3.2	2 per wk For 12 wk	EExt	1 (8–12)	8–12 RM	LWS	51.4 ± 10.9 vs 55.2 ± 10.2	3.8 (7.4)	≤0.05^c^	0.36
				EExt	3 (8–12)		MWS	45.6 ± 5.9 vs 48.3 ± 8.2	2.7 (5.9)	≤0.05^c^	0.38
Reid [24]	34	18–35	3 per wk For 8 wk	KFlex	1 (10–12)	3–18 RM	LWS	34.2 ± 6.4 vs 39.7 ± 8	5.5 (16.1)	≤0.05^c^	0.76
				KFlex	1 (15)		LWS2	40.4 ± 9.2 vs 42.8 ± 8.4	2.4 (5.9)	?	0.27
				KFlex	3 (6)		MWS	35.2 ± 5.3 vs 40 ± 5.6	4.8 (13.6)	≤0.01^a^	0.88
Reid [24]	34	18–35	3 per wk For 8 wk	KExt	1 (10–12)	3–18 RM	LWS	80.5 ± 15.8 vs 95.5 ± 17.8	15.0 (18.6)	≤0.01^a^	0.89
				KExt	1 (15)		LWS2	86.8 ± 5.8 vs 99.7 ± 7.7	12.9 (14.9)	≤0.05^c^	1.89
				KExt	3 (6)		MWS	90.0 ± 16.7 vs 103.6 ± 16.4	13.6 (15.1)	≤0.01^a^	0.82
Reid [24]	34	18–35	3 per wk For 8 wk	EFlex	1 (10–12)	3–18 RM	LWS	39.3 ± 4.2 vs 43.9 ± 6.3	4.6 (11.7)	≤0.01^a^	0.86
				EFlex	1 (15)		LWS2	39.2 ± 5.2 vs 47.1 ± 9.7	7.9 (20.2)	≤0.05^c^	1.02
				EFlex	3 (6)		MWS	42 ± 5.2 vs 45.5 ± 6.9	3.5 (8.3)	≤0.05^c^	0.57
Reid [24]	34	18–35	3 per wk For 8 wk	EExt	1 (10–12)	3–18 RM	LWS	28.5 ± 7 vs 35 ± 10.8	5.5 (22.8)	≤0.01^a^	0.71
				EExt^d^	1 (15)		LWS2	31.4 ± 4.8 vs 44.4 ± 5.5	13 (41.4)	≤0.01^a^	2.52
				EExt	3 (6)		MWS	33.4 ± 8.1 vs 40.3 ± 10.3	6.9 (20.7)	?	0.74
Reid [24]	34	18–35	3 per wk For 8 wk	SFlex^d^	1 (10–12)	3–18 RM	LWS	47.3 ± 10.7 vs 58.3 ± 10.7	11 (23.3)	≤0.01^a^	1.03
				SFlex	1 (15)		LWS2	48 ± 6.5 vs 55 ± 3.3	7 (14.6)	≤0.05^c^	1.36
				SFlex	3 (6)		MWS	52.9 ± 11.9 vs 64.4 ± 9.8	11.5 (21.7)	≤0.05^c^	1.05
Reid [24]	34	18–35	3 per wk For 8 wk	SExt	1 (10–12)	3–18 RM	LWS	48.2 ± 11.1 vs 54.8 ± 11.2	6.6 (13.7)	?	0.59
				SExt	1 (15)		LWS2	48.8 ± 8.7 vs 54.6 ± 10.6	5.8 (12.0)	?	0.60
				SExt	3 (6)		MWS	51.8 ± 9.1 vs 66.5 ± 11.1	14.7 (28.4)	≤0.01^e^	1.44
Sooneste et al. [25]	8	25.0 ± 2.1	2 per wk For 12 wk	SPC	1 (8)	8 RM	LWS	9.1 ± 1.6 vs 10.9 ± 2.5	1.8 (19.8)	≤0.05^c^	0.86
				SPC	3 (8)		MWS	9.1 ± 1.6 vs 11.9 ± 2.9	2.8 (30.8)	≤0.05^c^	1.20

? Data not available, *BC* bicep curl, *EExt* elbow extension, *EFlex* elbow flexion, *KExt* knee extension, *KFlex* knee flexion, *kg* kilograms, *LC* leg curl, *LWS* low weekly sets per exercise (≤5), *LWS2* low weekly sets per exercise data set two, *MWS* medium weekly sets per exercise (5–9), *N* number of subjects, *per wk* number of days trained per week, *Reps* repetitions, *RM* repetition maximum, *SD* standard deviation, *SExt* shoulder extension, *SFlex* shoulder flexion, *SPC* seated preacher curl, *y* years

^a^Significant differences between groups (*p* ≤ 0.01)

^b^Significantly greater than prior to training (*p* ≤ 0.05)

^c^Statistically significant difference compared with control group (*p* ≤ 0.05)

^d^Data excluded from analysis

^e^Significant differences between groups (*p* ≤ 0.01)

### Effects of Weekly Set Volume on Exercise-Specific 1RM

**Fig. 9:**
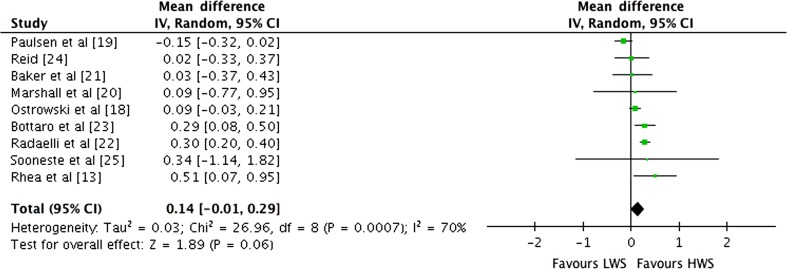
Forest plot of LWS vs HWS (MWS and HWS combined) on multi-joint and isolation exercise-specific 1RM by study. The *vertical dashed line* indicates the overall estimate of combined multi-joint and isolation studies mean effect size. *Horizontal lines* indicate 95% CI, *squares* indicate estimates, whereas square size is proportional to sample size, and *rhombus* indicates meta-analytically pooled estimates 95% CI. *1RM* one repetition maximum, *95% CI* 95% confidence interval, *HWS* high weekly sets per exercise (≥10), *IV* inverse variance, *LWS* low weekly sets per exercise (≤5), *MWS* medium weekly sets per exercise (5–9)

Outcomes for weekly sets categorised as LWS or HWS (MWS and HWS combined) for multi-joint and isolation exercise-specific 1RM by study are shown in the forest plot in Fig. 9. The pooled mean ES estimates of multi-joint and isolation strength data comprised 55 treatment groups from nine studies [13, 18–25]. There was moderate heterogeneity detected in the nine studies included in the meta-analysis of multi-joint and isolation weekly set outcomes (*I* ^2^ = 70%). When a random effects analysis was applied, a trivial effect was observed (ES 0.14; 95% CI −0.01 to 0.29; *p* = 0.06). Pre- to post-intervention strength gain was greater with HWS compared with LWS (ES difference 0.17). The mean ES for LWS was 0.80 (95% CI 0.47–1.13). The mean ES for HWS was 0.97 (95% CI 0.68–1.26). Examination of MWS versus HWS pre- to post-intervention strength differences was not feasible due to limited study data.

## Discussion

The purpose of this meta-analysis was threefold: (1) to investigate the effects of LWS, MWS or HWS of strength training on muscular strength per exercise; (2) to investigate if the magnitude of strength gain differs between multi-joint and isolation exercises; and (3) to provide a perspective on developing muscular strength across stages of training progression.

For novice and intermediate male trainees, the findings suggest that MWS and HWS strength training may lead to larger strength gains than LWS. For more experienced individuals, such as advanced and elite trainees, the sparse data available suggest that MWS and HWS strength training may create greater strength gains compared with LWS. The findings suggest that MWS and HWS strength training may produce marginally greater strength gains compared with LWS in certain contexts. LWS strength training per exercise appears to be less effective in both multi-joint and isolation exercises compared with MWS and HWS strength training.

The available data for exercise-specific 1RM provide support for the conventional view of a graded dose–response relationship where strength gains increase as a function of increased number of sets included in training. Examination of the effects of weekly set volume on exercise-specific 1RM support a graded dose–response relationship. The pooled mean ES estimate for LWS was 0.80 (95% CI 0.47–1.13) compared with HWS (0.97; 95% CI 0.68–1.26). The mean ES for LWS multi-joint-only exercises pre- to post-intervention was 0.81 (95% CI 0.65–0.97) compared with HWS (1.00; 95% CI 0.77–1.23). When LWS training was used as the control group and the HWS group was used as the experimental programme, a trivial ES of 0.18 (95% CI 0.01–0.34; *p* = 0.04) suggested that HWS training was more effective in producing strength gains. When LWS training was used as the control group and MWS training used as the experimental group, a trivial ES of 0.15 (95% CI 0.01–0.30; *p* = 0.04) suggested that MWS training is marginally more effective in producing strength gains. According to Cohen’s [17] classifications for ESs, ≤0.2, ≤0.5, ≤0.8 and ≥0.8 are considered trivial, small, moderate and large, respectively. The pre- versus post-strength training results for combined multi-joint exercises, multi-joint and isolation-only studies exposed a pre- to post-intervention graded dose–response relationship in strength gains. The data would thus suggest that MWS and HWS strength training produce marginally superior results compared with LWS.

The mean ES for isolation exercises on pre- to post-intervention strength gains in males was 0.95 (95% CI 0.30–1.60) for LWS compared with 1.10 (95% CI 0.26–1.94) for the HWS training programme. When LWS training was used as the control group and HWS training used as the experimental programme, a small ES of 0.23 (95% CI 0.06–0.40; *p* = 0.008) suggested that HWS training is more effective in producing strength gains for isolation exercises. The data for isolation exercises support the conventional belief in a graded dose–response pattern for an increased number of exercise sets and strength gain. Examination of the effects of weekly set volume on isolation exercise-specific 1RM was not feasible due to very small sample sizes that would lead to an over-parameterised model being performed on isolation exercises.

Examination of the effect of stage of training (beginner through elite) on strength gain was problematic as no available well controlled studies tested such a relationship. The limited available data suggested that comparable strength gains may be produced in earlier stages of training by multi-joint exercises when either MWS or HWS are employed. For advanced and elite trainees, the employment of either MWS or HWS may be considered as there was a small increase in strength in comparison with LWS training. The MWS and HWS pre- to post-strength programme produced marginally greater ESs compared with LWS training for the multi-joint and isolation combined exercise (ES 0.98, 1.01 vs 0.82, respectively). When LWS training was used as a control group and the HWS as the experimental programme, a trivial ES of 0.18 (95% CI 0.06–0.30; *p* = 0.003) was observed that suggested HWS training is more effective in producing strength gains.

### Current Meta-Analysis-Based Recommendations for Strength Development

The existing dogma on the number of sets best driving strength development has been largely indefinable and contentious. Set volume in RT has historically been an often-debated issue, based on varying recommendations favouring multiple-set programming with evidence cited from published meta-analyses [6–9]. However, these previous meta-analyses conducted on muscular strength gain reported inconsistent and varied outcomes. Of the four meta-analyses conducted [6–9] on this subject, none provided a clear and consistent trail of evidence identifying a dose–response relationship for maximum strength gains, upon which a determination of the best set scheme can be made. The inconclusiveness of previous research and previous meta-analyses have not altered the general preference in the programming of multiple sets over single-set training to create strength gains for every stage of training progression, beginner to elite.

Three of the four meta-analyses that are included within the ACSM [26] recommendations [6–8] have methodological constraints and the published evidence provided is disputed [11, 14]. Rhea et al. [6], in their meta-analysis, reported that significant differences emerged between the trained and the untrained groups. Rhea et al. [6] reported that 80% of 1RM with a training frequency of 2 days·week^−1^ and four sets per muscle group elicited superior gains in strength. In their consideration of untrained populations, they recommended RT loading at 60% of 1RM for 3 days·week^−1^ and employing four sets per exercise. Otto and Carpinelli [11] questioned the inclusion of studies in the Rhea et al. [6] meta-analysis and identified several confounding factors and inaccuracies that may have influenced the reliability of the meta-analysis. These included the reporting of incorrect ESs for advanced trainees, including claims that training at 80% 1RM resulted in an ES (1.8) that was three times greater than using 85% 1RM (ES 0.65). Furthermore, they reported that training each muscle group twice per week had an ES of 1.4 which was two times greater than training three times a week (ES 0.70). The inclusion of the Rhea et al. [13] study may have introduced error or bias as it reported an ES of 2.3 between groups in the bench press and an ES of 6.5 in the leg press when comparing the means of the one-set and three-set groups. The post-training standard deviation bench press results were two to three times superior to the pre-training standard deviation in both groups and the researchers did not provide confidence intervals for ES. This ES is almost three times larger than what is designated as statistically large (≥0.8) [27]. In addition, the leg press ES of 6.5 is more than eight times greater than a large ES, which presents an extraordinary ES that is not seen in any other related papers in the scientific literature [28]. As such, the inclusion of the Rhea et al. [13] leg press data in a meta-analysis could nullify the mean ES and spuriously affect the findings by increasing the heterogeneity of the meta-analysis and erroneously favouring multiple-set programming.

In the Peterson et al. [9] meta-analysis the authors propose that as strength increases so should RT volume. However, the evidence presented within their meta-analysis cannot be used to substantiate such a position. Inferences were made stating that competitive athletes should use eight sets per muscle group to promote strength gains. Such a conclusion is inconsistent with the evidence presented in their results, specifically the small number of ESs for eight sets. They present only six ESs contributing to the mean of the eight sets, and as such any conclusions warrant caution. Although not stated by the authors, the ESs presented could be derived from only one study. In contrast, the mean presented for four sets was accumulated from 199 ESs. Any conclusions drawn about the direct impact of eight sets compared with any other number of sets would be unreliable.

The meta-analysis of Wolfe et al. [7] sought to determine if the number of sets performed and the length of the RT programme affected outcomes. The subgroup analysis identified that programmes that lasted between 17 and 40 weeks did not generate significantly higher ESs in comparison with those lasting between 6 and 15 weeks. Significant interactions were reported for the set numbers and programme length, with multiple-set programmes producing superior increases in strength compared with single-set programmes (*p* ≤ 0.002). Data analysis indicated that trained individuals had greater increases in strength when using multiple-set programmes (*p* ≤ 0.001). Single-set programmes were proposed to be best suited to untrained individuals, as similar gains were noted with both single- and multiple-set programmes. These observations led the authors to suggest that as the subject’s progression in strength matures, there should be a concomitant change in programming from single to multiple sets to stimulate continuous strength gain.

Krieger’s meta-analytic review [8] comprised 14 studies (440 participants), with 30 treatment groups and a total of 92 ESs. The results showed that multiple sets were associated with a larger ES than a single set (difference 0.26 ± 0.05; 95% CI 0.15–0.37; *p* ≤ 0.0001). When the dose–response model was further analysed, there was a drift towards two to three sets per exercise compared with one set (difference 0.25 ± 0.06; 95% CI 0.14–0.37; *p* = 0.0001). No significant difference was reported between one set per exercise and four to six sets per exercise (difference 0.35 ± 0.25; 95% CI −0.05 to 0.74; *p* = 0.17) or between two to three sets per exercise and three to six sets per exercise (difference 0.09 ± 0.20; 95% CI −0.31 to 0.50; *p* = 0.64). The possibility of publication bias was assessed using methods described by Macaskill et al. [29]. Sensitivity analysis reported that no influential studies or publication bias were observed. This was performed by removing each study in turn to investigate the effect on the result of the multiple-sets variable. Krieger [8] concluded that two to three sets per resistance exercise was associated with 46% greater strength gains than one set in both trained and untrained subjects.

### Strengths and Limitations

There are several strengths of this meta-analysis that set it apart from previous analyses of set configurations. The strict inclusion criteria controlled for confounding variables when comparing the effects of LWS, MWS or HWS on strength outcomes. This meta-analysis also considered the potentially different effects of the use of isolation versus multi-joint (integrated) exercises on strength outcomes of the effects of LWS, MWS or HWS strength programmes. The design of this study also differed from others as it did not cluster outcomes; rather, data were pooled across strength measures to enhance external validity.

This analysis restricted its subject pool to male populations to better control for sex and endocrine influences. Factors that are known to affect strength include age, sex, physical activity levels, previous training status and endocrine status. Sex can influence muscle functioning and morphology [30, 31]. Men have reported greater muscle strength and size than women, due to higher levels of anabolic hormones and greater body size. The lower blood androgen levels of women have also been hypothesised to induce less relative muscle hypertrophy in response to RT compared with men [32]. However, several studies have failed to identify any difference between males and females with similar relative improvements in strength adaptations [33–35]. Tracey et al. [36] compared the hypertrophic response of the quadriceps of older men and women after nine weeks of training. Tracy et al. [36] reported that both male and females had an identical response of 12% in muscle volume. Conversely, results for upper body training have indicated differences in response to RT in men and women [35, 37]. Hubal et al. [38] assessed the variation in muscle size and strength in a large cohort of men and women (243 men, 342 women) after a 12-week unilateral RT programme targeting the non-dominant elbow flexor of the arm. Dynamic strength was assessed by determining the 1RM on the standard preacher curl exercise. Men and women exhibited wide ranges of 1RM strength gains from 0 to +250% (0 to +10.2 kg). In addition, men experienced 2.5% greater gains in cross-sectional area (*p* ≤ 0.05) compared with women. Regardless of men having greater absolute gains in strength, relative baseline strength increases in strength measures were greater in women compared with men (+25%).

Limited reliable data exist concerning the different levels of strength after RT programmes in men and women. The available data are from coefficients of variation (CV) of pre- and post-training strength measures. Some studies that analysed published means and standard deviations found equivocal strength variability in muscle size and strength for men and women [33, 39]. Equivocal data exist on whether there is an effect of sex or RT or an interaction effect between sex and RT. This may be due to issues concerning possible sex differences in variability, potentially due to small sample sizes. Previous studies found similarities in relative strength and size changes after RT [34, 40]. One factor that may explain these equivocal findings is the small sample sizes that limit the statistical power of these studies to detect significant differences between men and women.

As with previous studies, there were limitations driven by the shortcoming of primary data sources. Although the present meta-analysis endeavoured to include research papers from high-quality sources, the number of suitable studies was small and there remained differences in design and control among included studies. One of the nine included research papers used a randomised control design. The other eight did not include a control group; rather, they used a repeated measures design with baseline measure serving as the control, but baseline measures were not uniformly implemented across those studies. In this meta-analysis, the strength increases may be due to the repeated 1RM testing rather than other physiological adaptations. The exercise loading specificity of the 1RM-tested exercises may have impacted upon individuals’ performance. For example, a leg extension may have impacted upon the leg press performance, but not to the same degree as a leg press itself. Thus, the impact of specific RT loading versus non-specific exercises is accounted for in this analysis. Variation in programme order and the type of RT exercise between groups was not equivalent in all identified studies and this could impact upon set number and strength gain.

In addition, several sets of tested exercises versus nonspecific exercise can impact on an individual’s 1RM due to the ‘learning’ effect of the specifically tested exercise. This has been demonstrated by Dankel et al. [41] who conducted 1RM and maximal voluntary isometric contraction (MVC) testing on upper body isolation exercise (elbow flexion). One arm performed a 1RM test and MVC elbow extension exercises while the other arm performed 1RM test and MVC, in addition to three sets of exercises (70% 1RM) for 21 days. The results suggested that the increase in the trained subjects’ 1RM may not have been solely related to exercise volume, but was driven by the specificity of the exercise. These short-term adaptations may be due to performing the 1RM test rather than additional sets. The increase in subjects’ 1RM may have been due to a ‘learning effect’ caused by performing repeated testing sessions. This increase in strength, therefore, could be attributed to the principle of specificity as strength improvements may not be augmented by additional volume (sets). The studies by Ostrowski et al. [18] and Marshall et al. [20] included specific and nonspecific exercises that would, therefore, increase training volume. Ostrowski et al. [18] included one, two, or four sets per week of bench press with additional nonspecific assistance exercises, while Marshall et al. [20] included two, eight and 16 weekly sets of squats. The pre- to post-intervention increase in strength in some of the included isolation studies in this analysis may have been due to neurological crossover in the untrained contralateral arm. The results are applicable to isolation exercises involving smaller muscle groups as larger muscles may have different recovery patterns and properties.

### Future Development and Research

This meta-analysis demonstrates that potential outliers can affect pre- versus post-intervention strength data analysis [24] and may invalidate or skew the results when evaluating pre- to post-intervention strength difference. Previous observations lacking well controlled screening procedures that include unreliable evidence create difficulties for those attempting to summarise the existing data. The findings here suggest that researchers should be cautious when performing mixed model meta-analyses (mixed-sex subject groups), as this may produce spurious conclusions. There are limitations any time a comparison that combines subject characteristics (male–female or trained–untrained, for example) is conducted and the outcomes may or may not be valid. The body of scientific knowledge would be greatly improved if more RCT investigations were conducted on same age/sex and similar training status to clarify the set dose strength effects. This would help to establish the optimum set dose–response relationship and provide larger samples for meta-analyses, thus reducing the need to include low-power studies. As has been reported, meta-analyses have limitations when including the comparative outcomes of aggregated effects that do not necessarily assess the same construct [14]. Researchers to date have over simplified their RT designs and have inadvertently produced data that provide unreliable and confusing guidance regarding set numbers and strength gain. Sampling mixed-sex groups, use of expansive age ranges, use of multiple and different measurements, and the use of different training methods has resulted in a moderately large body of evidence that cannot fully answer the question at hand individually or collectively.

## Conclusion

Recommendations on the appropriate number of weekly sets per resistance exercise required to elicit desired strength gains are a divisive issue for researchers, clinicians and fitness trainers. Within each occupational group, there are those that dogmatically hold that an effect of LWS training is best for creating strength and there are those that ferociously defend HWS strength training per exercise as the best means of gaining strength. The current literature does little to settle this argument and the optimal sets-to-strength gain relationship remains unquantified. The ACSM [10], in a position stand statement presented to the public as authoritative, has added to the confusion because it is based on limited evidence and oversimplified recommendations.

The current meta-analysis presents additional evidence regarding a graded dose–response relationship between sets and strength. The prescription of RT for strength gains is a complex process of manipulating programme variables. A known reference relationship between sets and strength gain would be invaluable to every practitioner whether they are clinic or gym based. This research project analysed a limited set of available data and cautiously advocates the use of MWS for beginners, novice trainers, or the time-dependent trainer. For well-trained individuals, the use of either MWS or HWS strength training may be appropriate. These more advanced trainees may benefit from additional time and training volume to reap the smaller increases in performance normally seen at this level of training progression. A consideration with this advanced group is the interaction of additional strength training volume and time with the achievement of other fitness goals. For those trainees in the middle ground, not a novice and not advanced, the existing data provide a relationship between weekly sets and strength gain, as a graded response to additional weekly sets produced increases in pre- versus post-intervention strength. This suggests that MWS or HWS strength training is appropriate for this group. It is very apparent that more investigations and replication studies using appropriate study designs and comparable subject samples are required.
